# Outer Retinal Assessment Using Spectral-Domain Optical Coherence Tomography in Patients With Alzheimer's and Parkinson's Disease

**DOI:** 10.1167/iovs.17-23240

**Published:** 2018-06

**Authors:** Atsuro Uchida, Jagan A. Pillai, Robert Bermel, Aaron Bonner-Jackson, Alexander Rae-Grant, Hubert Fernandez, James Bena, Stephen E. Jones, James B. Leverenz, Sunil K. Srivastava, Justis P. Ehlers

**Affiliations:** 1 Ophthalmic Imaging Center, Cole Eye Institute, Cleveland Clinic, Cleveland, Ohio, United States; 2 Cole Eye Institute, Cleveland Clinic, Cleveland, Ohio, United States; 3 Department of Neurology, Cleveland Clinic, Cleveland, Ohio, United States; 4 Lou Ruvo Center for Brain Health, Cleveland Clinic, Cleveland, Ohio, United States; 5 Mellen Center for Multiple Sclerosis, Cleveland, Ohio, United States; 6 Center for Neurological Restoration, Cleveland, Ohio, United States; 7 Department of Quantitative Health Sciences, Lerner Research Institute, Cleveland, Ohio, United States; 8 Imaging Institute, Cleveland, Ohio, United States

**Keywords:** Alzheimer's disease, neurodegenerative disease, Parkinson's disease, OCT

## Abstract

**Purpose:**

To investigate outer retinal parameters among patients with various chronic neurodegenerative disorders by using spectral-domain coherence tomography (OCT) in a prospective cross-sectional cohort study.

**Methods:**

A total of 132 participants were enrolled following a comprehensive diagnostic evaluation with neurologic, neuropsychology, and magnetic resonance imaging volumetric evaluations. Participants were 50 years or older, either diagnosed with Alzheimer's disease (AD) dementia, amnestic mild cognitive impairment (MCI), non-AD dementia, Parkinson's disease (PD), or age- and sex-matched controls. All participants underwent a macular cube scan for both eyes by using the Cirrus 4000 HD-OCT (Zeiss, Oberkochen, Germany). The OCT image with the best quality was selected for further analysis. Outer retinal parameters including ellipsoid zone mapping and outer nuclear layer metrics were evaluated with a novel software platform.

**Results:**

One hundred twenty-four eyes of 124 participants with AD dementia (24 eyes), amnestic MCI (22 eyes), non-AD dementia (20 eyes), PD (22 eyes), and age- and sex-matched controls (36 eyes) were included in the analysis. Eight eyes were excluded either due to the presence of macular disease or poor quality of the OCT image. The mean ages of participants were 65.9 ± 8.9 years. The outer retinal thickness measures did not show any statistical significance between the groups. However, ellipsoid zone to retinal pigment epithelium volume correlated with cognitive testing scores in all study participants.

**Conclusions:**

There were no identifiable differences in the outer retinal metrics across neurodegenerative disease groups and controls. The relationship between the degree of cognitive impairment and ellipsoid zone to retinal pigment epithelium volume warrants further study.

Major central nervous system disorders have been reported to present ocular manifestations that reflect the condition of the brain.^1^ With the advent of optical coherence tomography (OCT) that enables direct noninvasive retinal imaging with an assessment of subtle changes in the retina, it has become of increasing interest to use the retina as a potential surrogate for the study of chronic neurodegenerative disorders of the brain, such as Alzheimer's disease (AD) or Parkinson's disease (PD).

AD is a progressive neurodegenerative disorder and is the most common cause of dementia in the elderly population.^2^ In AD, visual symptoms are often among the earliest manifestations.^3,4^ Histologic studies indicate an abnormal accumulation of amyloid-β (Aβ) and phosphorylated tau in the retina, similar to neuropathologic features of the disease in the brain.^5^ In addition, retinal ganglion cell (RGC) loss and retinal nerve fiber layer (RNFL) atrophy have been previously reported.^6,7^ Mild cognitive impairment (MCI) is a state which manifests impairment of cognitive functions, with the otherwise normal performance of activities of daily living. MCI lies on a spectrum between normal cognitive aging and dementia and is recognized as a prodromal phase for AD dementia in many cases.^8,9^ The amnestic type (amnestic MCI) shows the highest annual incidence conversion rate to AD dementia.^10^

PD is a chronic and progressive neurodegenerative disorder classically associated with motor dysfunction.^11^ Major pathologic changes result primarily from the apoptotic loss of the dopaminergic neurons in the substantia nigra associated with dopaminergic deficiency.^1^ Accumulating evidence suggests that dopamine is an important neurotransmitter for visual processing in the retina.^11,12^ PD often involves ocular manifestations such as diminished visual acuity, deteriorated contrast sensitivity, or visual hallucinations.^1,13^ Recent histologic studies demonstrated the presence of a misfolded and phosphorylated α-synuclein protein, which is a pathologic hallmark of PD, in the inner retina.^14,15^

Over the last decade, OCT has been used to assess the retinal thickness in patients with AD, MCI, and PD.^16–20^ Previous studies have mainly focused on inner retinal alterations and have shown that reduction in RNFL thickness as measured by OCT is a potential early biomarker for axonal loss or neuronal death.^16–29,21^ There have been few published reports investigating the outer retinal features/metrics in these conditions by using OCT; although, swelling of cone photoreceptors have been reported in patients with dementia with Lewy bodies,^22^ a disease which shares the same underlying biologic changes in the brain with PD dementia. The aim of this study was to quantitatively assess the outer retinal alterations observed in various neurodegenerative diseases (AD, MCI, non-AD dementia, and PD) with OCT.

## Subjects and Methods

This was a prospective cross-sectional cohort study approved by the Cleveland Clinic Institutional Review Board. The study was conducted in accordance with the declaration of Helsinki. A total of 132 participants evaluated at the Cleveland Clinic Lou Ruvo Center for Brain Health or Center for Neurological Restoration between October 2012 and August 2014 were included in the study. Participants were either diagnosed with AD, amnestic MCI, non-AD dementia, or PD and were age- and sex-matched with normal cognition controls. All participants were 50 years or older, and informed written consent was obtained before enrollment in the study. Participants underwent screening by a clinician for a history of ophthalmic disease. Each participant was screened for systemic diseases, such as diabetes and hypertension. Exclusion criteria included any OCT abnormalities that might impact retinal layer thickness (e.g., glaucoma, macular degeneration, diabetic retinopathy, and epiretinal membrane) or insufficient OCT image quality for interpretation.

### Neurologic Clinical Diagnostics and Evaluation

All participants underwent comprehensive neurologic and ophthalmic examinations, as mentioned previously.^20^ A neurologic evaluation, detailed cognitive testing, and magnetic resonance imaging (MRI) of the brain were performed for each participant. The diagnosis of each neurologic and cognitive disorder were made by an experienced neurologist. The National Institute on Aging/Alzheimer's Association 2011 diagnostic criteria was used at consensus multidisciplinary case conferences to establish diagnosis of AD (probable AD with evidence of the AD pathophysiologic process; evidence of MRI hippocampal and medial temporal atrophy), amnestic MCI (MCI due to AD-intermediate likelihood), non-AD dementia (dementia unlikely due to AD), and normal cognition.^23,24^ Participants were classified as having PD by using the United Kingdom Parkinson's Disease Society Brain Bank clinical diagnostic criteria.^25^ For patients with PD, the Unified Parkinson's Disease Rating Scale (UPDRS) was administered in the medication-off state. As part of the neurocognitive assessment, study participants completed the Montreal Cognitive Assessment (MoCA),^26^ the Logical Memory subtest of the Wechsler Memory Scale Fourth Edition,^27^ the Hopkins Verbal Learning Test Revised,^28^ phonemic and semantic verbal fluency, and the Trail Making Test (parts A and B).^29^ The MoCA is a rapid screening tool for mild cognitive impairment.^26^ The test (a perfect score of 30 points and the worst score of 0) can be administered in approximately 10 minutes. It allows assessment of multiple cognitive domains including attention, concentration, working memory, short-term memory, verbal fluency, visuospatial ability, conceptual thinking, and orientation to time/place. One point is added for subjects with little or no formal education. Scores of 25 or below are considered abnormal. The UPDRS is a comprehensive rating system design to assess the severity of PD from both motor and nonmotor symptoms. It is widely used as a clinical evaluation tool for monitoring disease progression and treatment response. The test is made up of four sections with 42 items (most items are scaled from 0 to 4, with 4 representing the greatest level of dysfunction) and is evaluated by an interview and clinical observation. Part III, the motor examination section which include items such as speech, facial expression, postural stability, and rest tremor amplitude, is considered particularly useful to evaluate the longitudinal progression of the disease, and is used in this study (part III is graded between 0 to 108 points).

### Ophthalmic Imaging Assessment

**Figure 1:**
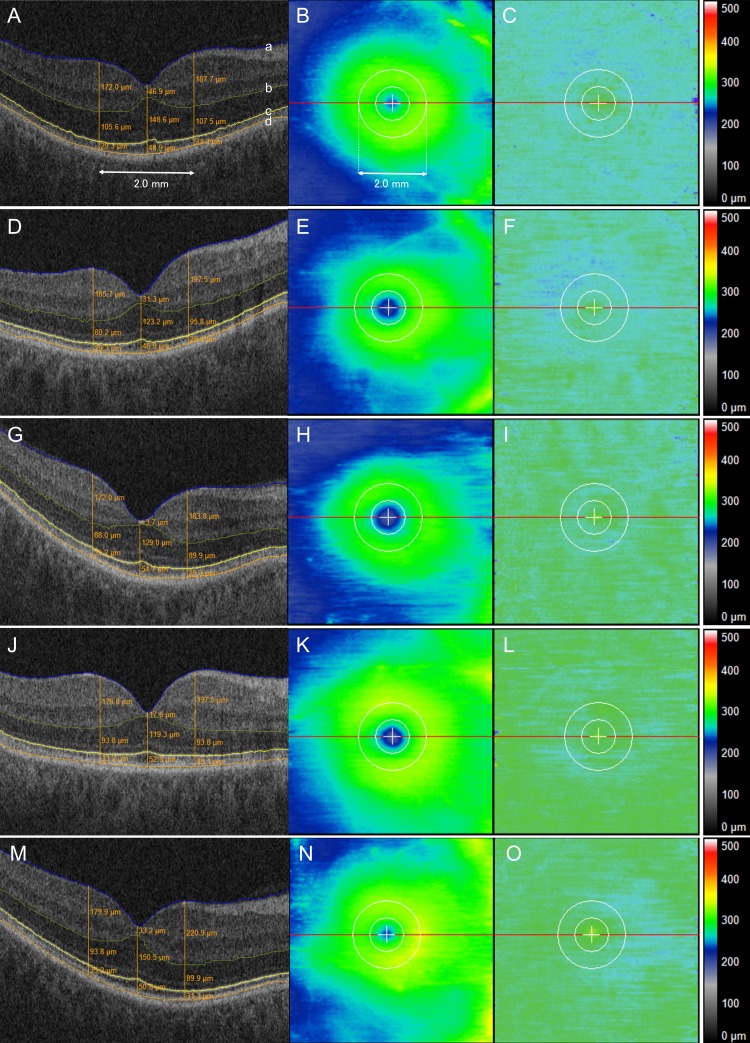
(A–C) An example of SD-OCT images of the retina in a patient with AD dementia (case 106, 58-year-old male, right eye, MoCA score 15 points, cluster A). (A) Horizontal B-scan of the posterior pole crossing the central fovea. The segmentation software identifies the boundaries of retinal layers, and each boundary is corrected manually by an experienced physician in a blinded fashion. The ILM (a), the outer boundaries of the OPL (b), EZ (c), and the inner boundaries of the RPE (d). For retinal thickness measurement, three orange lines parallel to the vertical axis of the OCT image are illustrated at the fovea, 1.0 mm nasal and 1.0 mm temporal to the fovea. Orange figures buried in retinal layers indicate retinal thickness between the neighboring boundaries. (B) En face retinal thickness map (ILM to RPE) of the same patient. The outer circle around the fovea is 2.0 mm in diameter. (C) En face retinal thickness map (EZ to RPE). (D–F) An example SD-OCT image of a patient with amnestic mild cognitive impairment (case 11, 73-year-old male, right eye, MoCA score 22 points, cluster C). (G–I) A patient with non-AD dementia (case 14, 70-year-old male, right eye, MoCA score 12 points, cluster A). (J–L) A patient with PD (case 18, 58-year-old male, right eye, MoCA score 29 points, UPDRS motor score 18 points, cluster B). (M–O) An example of SD-OCT images of age- and sex-matched controls with normal cognition (case 96, 60-year-old female, right eye, MoCA score 26 points, cluster B).

All participants underwent a macular cube scan using the Cirrus 4000 HD-OCT (Zeiss, Oberkochen, Germany) with the 512 × 128 (horizontal × vertical) scan pattern. Each raster scan covered a nominal 6 × 6-mm area centered at the fixation point in the posterior pole. OCT scans were performed for both eyes by an experienced technician. All OCT scans were reviewed for image quality and analyzed by a masked experienced retina specialist. For each participant, an OCT image with the best quality was selected for further analysis and was excluded if the scan was of insufficient quality for segmentation analysis. Retinal thickness/volume between the internal limiting membrane (ILM) to retinal pigment epithelium (RPE), outer nuclear layer (ONL) to ellipsoid zone (EZ), and EZ to RPE were measured using a novel OCT layer and pathology analysis software program, as previously described (Fig. 1).^30^ ONL was defined as the outer boundaries of outer plexiform layer (OPL) to EZ band. Semiautomated segmentation was performed using the software platform to identify the boundaries of interest. Line-by-line validation with manual correction was carried out by an expert reviewer for all scans by using a stylus-based input device (Wacom Tech Corp., Saitama, Japan). Following segmentation, the various retinal volumes and thicknesses were exported for analysis. For thickness measurements, mean foveal thickness and juxtafoveal thickness (i.e., 1.0 mm nasal or temporal to the fovea) measurements were taken of each metric.

### Statistical Analysis

The statistical analysis was performed using R software (Vienna, Austria).^31^ A case-control analysis was undertaken, with the thickness/volume OCT metrics collected with age- and sex-matched controls. The outer retinal thickness (ONL to RPE) was reported with a mean ± SD of 115 ± 8 μm.^32^ If the difference in the experimental and control means is estimated as 10 μm with an SD of 8 μm, 11 participants are required in each group to be able to reject the null hypothesis (an alpha set at 0.05 and power set at 0.8). The Kolmogorov-Smirnov test was used to evaluate the normality of sample distribution, and the Bartlett test was used to assess variance homogeneity. One-way ANOVA models with posthoc Bonferroni multiple comparison procedures were used to compare groups on age and OCT measures. The Kruskal-Wallis 1-way ANOVA on ranks with posthoc Bonferroni adjustments were used to compare cognitive test results. The chi square test was used to compare sex and laterality of the eye. The unpaired Student's *t*-test and the Mann-Whitney *U* test were used to compare outcome variables between the patient and control groups. Data are presented as the mean ± SD in the characteristic of participants section and mean ± SE in identified statistical measures. In all statistical analyses, *P* < 0.05 was considered statistically significant.

## Results

### Clinical Characteristics and Demographics

Overall, 132 eyes of 132 participants were enrolled in the study. Eight eyes were excluded from the study either due to the presence of macular degeneration (1 eye), epiretinal membrane (2 eyes), vitreomacular traction syndrome (1 eye), or limited OCT image quality (4 eyes). The number of eyes excluded from each group was 2 (8%) eyes in AD dementia, 3 (12%) eyes in amnestic MCI, 2 (9%) eyes in non-AD dementia, no eyes in PD, and 1 (3%) eye in age- and sex-matched controls (*P =* 0.346). The remaining 124 eyes of 124 participants included AD dementia (24 eyes), amnestic MCI (22 eyes), non-AD dementia (20 eyes), PD (22 eyes), and age- and sex-matched controls (36 eyes) by the frequency matching sampling design. The mean ages of participants was 65.9 ± 8.9 years. Clinical demographics of the participants are shown in Table 1. There was no statistically significant difference in age and sex between the groups.

**Table 1:**
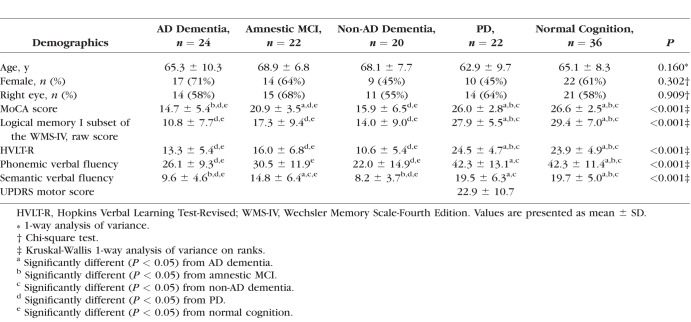
Patient Demographics

### Spectral Domain OCT (SD-OCT) Findings

**Figure 2:**
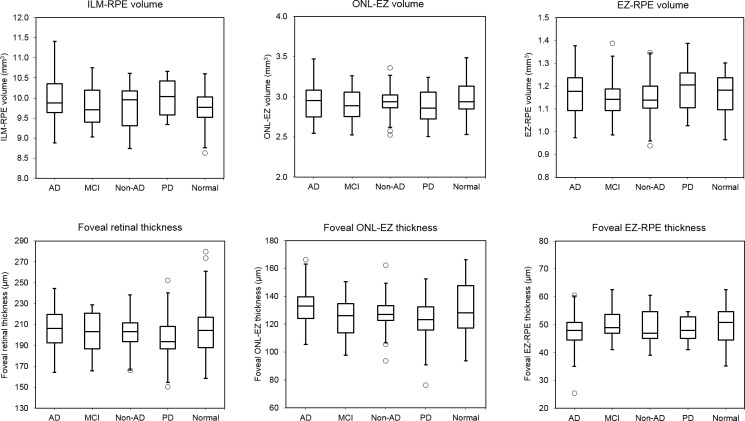
Box plot reporting retinal measures of each group. Middle horizontal line inside box indicates median. The bottom and top of the box are the 25th and 75th percentiles, respectively. The ends of the whisker show the minimum and maximum for each group. Circles represent outliers, defined as values over 1.5 box lengths from either end of the box.

The data in Table 2 represent retinal metrics by analysis group. The box plot in Figure 2 illustrates median, 25th/75th percentile, and lower/upper data within 1.5 interquartile ranges of each retinal metric. The mean foveal ONL-EZ thickness in AD dementia, amnestic MCI, non-AD dementia, PD, and the control group was 131.7 ± 2.8, 124.2 ± 3.0, 127.7 ± 3.0, 123.4 ± 3.2, and 131.0 ± 2.9, respectively (*P =* 0.210); the mean foveal EZ-RPE thickness was 48.1 ± 1.6 μm, 50.1 ± 1.1 μm, 48.7 ± 1.4 μm, 47.7 ± 1.0 μm, and 49.2 ± 1.3 μm, respectively (*P =* 0.755). The area coverage or the proportion of EZ-RPE attenuation evaluated as EZ-RPE thickness less than 20 μm was below 1% in all groups (*P =* 0.624). Similarly, the area coverage of RPE atrophy evaluated as EZ-RPE thickness equaling zero also did not show any statistical significance between the groups (*P =* 0.659). After all, none of the OCT measures, including ILM-RPE, ONL-EZ, or EZ-RPE thickness/volume, were significantly different across all groups.

**Table 2:**
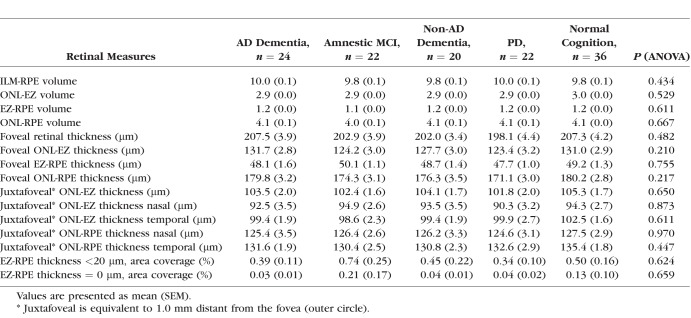
Retinal Metrics by Analysis Group

### Correlation Between OCT Retinal Variables and Cognitive Measures

**Figure 3:**
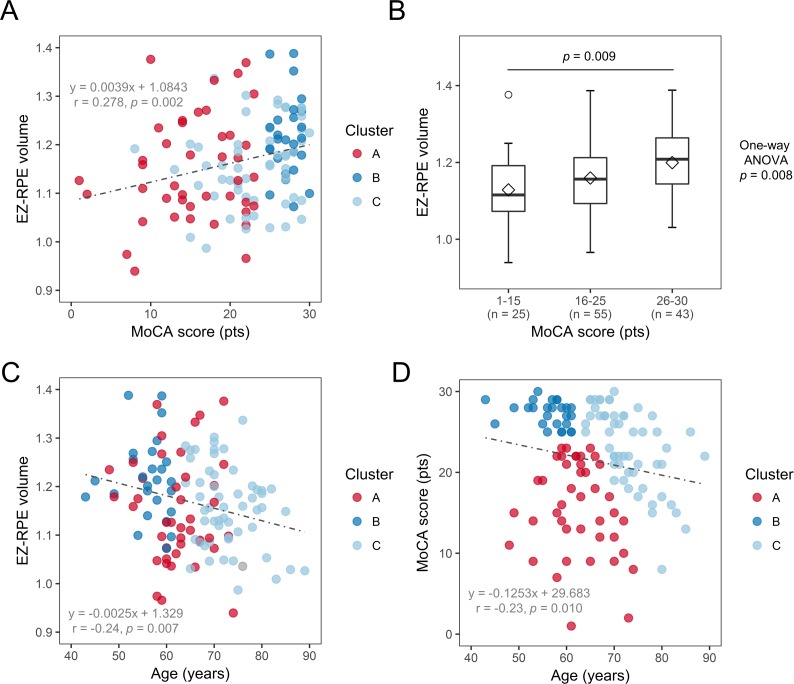
(A) Scatter plot showing a weak positive correlation between MoCA score and EZ-RPE volume (Spearman's rank correlation coefficient r = 0.278, P = 0.002). Color markers represent each case in cluster A, B, and C. (B) Box plot reporting EZ-RPE volume of three groups depending on MoCA score (0 to 15, 16 to 25, and 26 to 30 points). Box plot is presented in the same manner as in Figure 2 (marker in the box represents the mean of each group). The mean EZ-RPE volume appears incremental from low to high MoCA score group. (C) Scatter plot showing a weak negative correlation between age and EZ-RPE volume (Pearson's correlation coefficient r = −0.24, P = 0.007). (D) Scatter plot showing a weak negative correlation between age and MoCA score (Spearman's rank correlation coefficient r = −0.23, P = 0.010).

We further investigated whether there was any correlation between OCT retinal variables and cognitive measures in all study participants. There was a weak correlation between EZ-RPE volume and several cognitive measures including MoCA score (Table 3; Fig. 3A, Spearman's rank correlation coefficient 0.278, *P =* 0.002). When all study participants were divided into 3 groups depending on the MoCA score, the mean EZ-RPE volume appeared incremental from low to high MoCA score group (Fig. 3B). However, age was considered a potential confounding factor, because age was correlated with both EZ-RPE volume and MoCA score (Fig. 3C, 3D). Multiple linear regression analysis revealed that both age (unstandardized coefficient, −2.04 × 10^−3^ [95% confidence interval −3.89 × 10^−3^ to −2.80 × 10^−4^]; *P =* 0.032) and MoCA score (unstandardized coefficient, 3.39 × 10^−3^ [95% confidence interval 8.58 × 10^−4^ to 5.92 × 10^−3^]; *P =* 0.009) were independent, influential factors on EZ-RPE volume (Table 4).

**Table 3:**
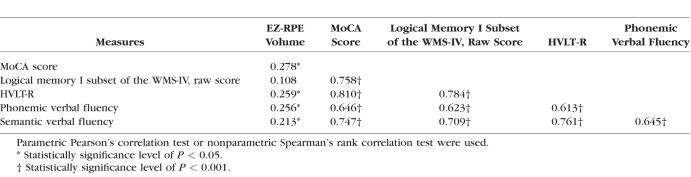
Correlation Coefficient Matrix of EZ-RPE Volume and Cognitive Measures in All Study Participants

**Table 4:**
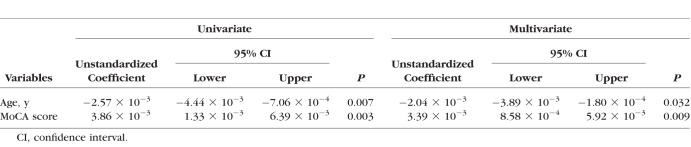
Univariate and Multivariate Linear Regression Analysis on EZ-RPE Volume in All Study Participants

Subsequently, exploratory cluster analysis was performed to find clinical characteristics of cases with decreased EZ-RPE volume. Hierarchical clustering (Wald's methods, squared Euclidean) was used to separate the eligible 124 cases into three clusters (groups); variables included age, MoCA score, and EZ-RPE volume. One case without a MoCA score was excluded from this analysis. Results are shown in Table 5. Cluster A, B, and C included 42, 26, and 55 cases, respectively. Cluster B was youngest (mean age, 56.2 years), MoCA score was highest (mean, 27.3 points) among the three clusters, and EZ-volume was greatest (mean, 1.22). Cluster A was older than cluster B (mean age, 62.4 years), with a significantly lower MoCA score (mean, 15.6 points). In addition, cluster A EZ-RPE volume was significantly lower than cluster B but comparable to cluster C. Cluster C was the oldest (mean age, 72.9 years), the MoCA score (mean, 23.2 points) was lower than cluster B but higher than cluster A, and EZ-RPE volume was significantly lower than cluster B. Regarding the number of study participants from the diagnostic groups, cluster A mainly consisted of AD (38%), MCI (19%), and non-AD dementia (29%) patients. Meanwhile, cluster B mainly consisted of PD (38%) and age- and sex-matched controls (46%). Cluster C consisted almost equally of the four neurodegenerative diseases (range, 13−28%), with the largest number of age- and sex-matched controls (38%). Because the difference of EZ-RPE volume between cluster A and B is better explained by the difference in MoCA score (estimate using coefficients from multiple linear regression analysis, 3.39 × 10^−3^ × [27.3 − 15.6] = 0.040) than the difference in age (−2.04 × 10^−3^ × [56.2 − 62.4] = 0.013), cluster A may be termed as “a subset of cases with decreased EZ-RPE volume more linked to low MoCA score than age.” On the contrary, the difference of EZ-RPE volume between cluster B and C is better explained by the difference in age (−2.04 × 10^−3^ × [56.2 − 72.9] = 0.034) than the difference in MoCA score (3.39 × 10^−3^ × [27.3 − 23.2] = 0.014), therefore, cluster C may be termed as “a subset of cases with decreased EZ-RPE volume more linked to older age than MoCA score.”

**Table 5:**
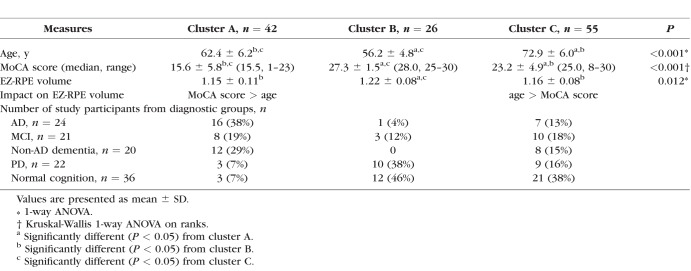
The Results of Cluster Analysis for Three Major Clusters Identified in Simulation

## Discussion

The retina is an extension of the central nervous system, derived from an outpouching of the diencephalon during embryonic development.^1^ Because the retina and brain share similarities and retinal layers are accessible for noninvasive diagnostic testing with OCT, the ability to quantify neuronal death or alterations in retinal structure is an attractive biomarker for evaluating various neurodegenerative diseases.^1^ Recent research has focused on early detection of retinal morphologic alterations with OCT so that treatment intervention can be initiated in the early stages. In fact, some investigators have suggested that the RNFL defects may be the earliest signs of AD, even prior to damage to the neurons in hippocampus.^33^

Our study is unique in that we analyze the volumetric and thickness parameters of the outer retina. A few studies have analyzed the outer retinal thickness of the macula in PD, but with inconsistent results. Spund et al^34^ investigated 30 eyes in patients with PD and identified that the foveal pit was thinner and broader on SD-OCT in PD. However, no difference was found in photoreceptor thickness in the central fovea.^34^ Meanwhile, Garcia-Martin et al.^17^ studied 129 eyes in patients with PD, and retinal layer segmentation with SD-OCT revealed reduced OPL thickness in PD. Roth et al.^35^ performed retinal layer segmentation in 68 patients with PD and found that combined ONL and photoreceptor layer thickness were significantly reduced in patients with PD compared with healthy controls (118.6 vs. 123.5 μm, *P =* 0.001). In this study, we did not find any statistical difference in outer retinal thickness or outer retinal volume across our study groups. These discrepancies between studies could be due to differences in sample size, the clinical stage of the disease, or the OCT systems/software used in the study. The segmentation software platforms vary across OCT systems and this must be considered, particularly if conventional segmentation systems are used.

A postmortem study demonstrated that retinal dopamine content was reduced in PD patients without levodopa therapy.^36^ In the retina, the largest source of neurotransmitter dopamine is dopaminergic amacrine cells located in the inner nuclear layer and inner plexiform layer,^37^ and dopamine act on all the main cell types in the outer and inner layers of the retina.^38,39^ Photoreceptors receive paracrine input of dopamine via D2 receptors on the outer segment.^38,39^ Dopamine input reduces melatonin production in photoreceptors, thereby affecting the circadian rhythm of the retina.^38,39^ Also, dopamine may regulate the phototransduction cascade and other cellular functions of photoreceptors such as outer segment disc shedding.^38–40^

Given the potential impact of the neurotransmitters in the retina, one key question is whether photoreceptors are morphologically affected in PD or other neurodegenerative diseases. Roth et al.^35^ speculated that reduced outer retinal thickness might be attributed to transsynaptic degeneration of ONL neurons as a result of impaired synaptic input. However, dopaminergic amacrine cells are not considered to form synaptic terminals with photoreceptors. Dopamine is released from free terminals into the extracellular space, diffusing across to reach the outer retinal layer.^38,39^ In addition, the potential loss of photoreceptors due to impaired mutual interactions with dopaminergic amacrine cells, which are crucial for maintaining the circadian rhythm of the retina, has been proposed.^35^ However, as shown in animal studies, dopamine is not required to keep the circadian rhythm of melatonin production, because circadian rhythmicity is generated by photoreceptors.^41^ Additionally, photoreceptor survival or morphology was not affected in D4 receptor knockout mice (D4 receptor is a D2-family receptor on photoreceptors in mice).^40^ In one study, Devos et al.^42^ reported that there were no differences in ERG in the PD group compared with controls. Overall, these data suggest that retinal dopamine deficiency does not significantly impact photoreceptor survival in PD.

In AD, histologic studies of humans postmortem and animal models have disclosed a deposition of Aβ in the posterior segment of the eye, mainly localized to the inner retina and optic disc.^5,43^ Aβ might be particularly neurotoxic for RGCs, because increased TUNEL-positive cells were seen in GCL, highlighting enhanced apoptosis in the inner retina, but not in the outer retina, in a mouse model of AD.^44^ It is hypothesized that neurotoxic Aβ deposition may cause persistent synaptic transmission defects, dendritic network remodeling of cholinergic amacrine cells, and insufficient acetylcholine levels.^45^ Currently, it is assumed that RNFL thinning in AD may be a consequence of retrograde degeneration of the RGC axons occurring in the cortical regions^16,18^; however, some investigators hypothesized that RNFL reduction might be partially ascribed to the hypoxic state of the retina caused by the narrowing of retinal blood vessels and reduced retinal blood flow rate, which has been observed in AD.^46^ Animal models have supported this idea through the identification of microvascular Aβ deposition within the retina.^44^ In addition, Aβ was identified as diffusely deposited in the retinal vascular network, but less focally deposited in choroidal vessels in an aging mouse model.^47^ In this study, we did not find any significant difference in ONL-RPE thickness in the AD group compared with other groups. Because outer retinal layers are mainly oxygenized by choroidal vessels, it is possible that the outer retina may be more tolerant to a retinal vessel narrowing-induced hypoxic state compared with the inner retina in AD.

Although no significant difference in OCT retinal variables was found across the neurodegenerative diagnostic groups, interestingly, EZ-RPE volume was correlated with multiple cognitive measures including MoCA score. The results from the cluster analysis indicate that there were subset of study participants (cluster A) whose EZ-RPE volume and MoCA score were significantly decreased at relatively younger age. It is possible that patients in cluster A, having a common severe cognitive impairment due to AD, MCI, and non-AD dementia, might share an unknown genetic background. Moreover, a small number of PD patients and age- and sex-matched controls in cluster A might be in a preclinical condition of severe cognitive impairment. Furthermore, measurement of EZ-RPE volume with SD-OCT may be used as a potential biomarker for severe cognitive impairment, which will be highly valuable given the fast image acquisition and noninvasive nature of SD-OCT. The pathologic mechanism behind why EZ-RPE volume decreased in low MoCA score patients (cognitive impairment or dementia regardless of the underlying diagnosis) is beyond the scope of this study and requires further investigation. It might be somewhat related to the fact that outer segments are mostly composed of phospholipids. Because the amount of decrease in EZ-RPE volume was clinically marginal, we assume that it might be a functional abnormality of the photoreceptors (e.g., shortening of outer segments due to decreased production of discs or slowed turnover of disc shedding), rather than structural abnormalities, or apoptosis of the photoreceptors. Observation of photoreceptors using adaptive optics in low MoCA score individuals may give some clue to answer this question.

This study has several advantages over previous studies. As mentioned in our earlier report, all participants met National Institute on Aging/Alzheimer's Association criteria for AD dementia and MCI due to AD.^20^ Participants with PD and normal cognition, along with AD and MCI participants, also underwent detailed neurocognitive testing and MRI volumetric scans to accurately evaluate clinical characteristics. The high-resolution SD-OCT system should be evaluated when comparing the results to other previous studies using time-domain OCT systems. Moreover, all the B-scan OCT images were inspected for image quality and the semiautomatically segmented retinal layers were manually corrected by a single experienced physician in a masked fashion. In our review of the literature, this is also the first study to examine outer retinal metrics in volumetric fashion.

As with any other cross-sectional study, there are several limitations that should be acknowledged. One of the main limitations of the present study is the absence of longitudinal follow-up. In addition, our study is limited by a relatively small sample size. As for OCT image analysis, some images were tilted in cross-sectional images due to the horizontally eccentric positioning of the OCT scanning beam within the pupil.^48^ In fact, 27 (22%) eyes were tilted equal to or more than 20 degrees, an angle of incidence measured with ImageJ software (version 1.51j8; National Institutes of Health, Bethesda, MD, USA) in a similar method reported by Hariri et al.^48^ The number of eyes with tilted OCT images (of equal to or more than 20 degrees) in each diagnostic group was 4 (17%) eyes in AD, 2 (9%) eyes in MCI, 4 (20%) in non-AD dementia, 4 (18%) eyes in PD, and 13 (36%) eyes in age- and sex-matched controls. It is reported that retinal thickness increases and correlates with the degree of tilting.^48^ Because the retinal thickness measurement was performed parallel to the vertical axis of the OCT image and not perpendicular to the retinal surface or RPE in our study, it is possible that retinal thickness may be slightly overestimated, and/or the location of the measurement may be displaced relative to the fovea, leading to broadened coverage on cross-sectional OCT.^48^ To address this issue, meticulous attention is required during OCT exam to obtain horizontally oriented B-scans by intentionally encouraging subjects to gaze nasally or temporally. Also, some investigators indicate that accurate measurement of OPL or ONL thickness is challenging due to the optical properties of Henle's fiber layer. These fine axons from the fovea occupy two-thirds of the OPL thickness, based on anatomy. They are affected significantly by the positioning of the scanning beam on the pupil, typically are not visualized on standard OCT images, and are typically included within the ONL when visualizing the OCT B-scan due to the optical properties.^49–51^ Therefore, the ONL-EZ thickness may be overestimated in this study due to the inclusion of the invisible Henle's fiber layer in the ONL in some of our participants. Additionally, the OCT scan pattern used in our study was 512 × 128 scan covering a 6 × 6-mm area, which yields a denser assessment horizontally than vertically. Since a shallower foveal slope in PD occurs vertically a greater degree than horizontally,^52^ reduced vertical sampling in our study may have impacted the results. Moreover, we did not assess the axial length of each patient, implicating that high myopic eyes might have been inadvertently included in our study. Eyes with long axial length are known to produce magnification errors of retinal thickness measurement.^53^ Furthermore, the retinal variables defined in our study may have been too thick to detect the difference or a direct linear/areal/volumetric comparison may not be sufficient to distinguish neurologic disorders.^54^ Development of a mathematic model by using a combination of parameters may allow the differentiation of neurodegenerative disease from healthy controls.^54^ Finally, some of the patients may not be affected severely enough for retinal changes.

In conclusion, macular volumetric and linear SD-OCT findings suggested that the outer retinal thickness measures analysis did not show any statistical significance between the neurodegenerative disease groups and controls. However, EZ-RPE volume was significantly associated with MoCA score in all study participants, indicating a potential biomarker for detecting severe cognitive impairment. Although AD and PD pathology may be a much smaller magnitude or extent in the outer retina than in the inner retina or the brain, further longitudinal study is warranted to confirm these observations.
